# Effect of Moderate-Intensity Exercise on Plasma C-Reactive Protein and Aortic Endothelial Function in Type 2 Diabetic Mice

**DOI:** 10.1155/2010/149678

**Published:** 2010-08-02

**Authors:** Nada Sallam, Majid Khazaei, Ismail Laher

**Affiliations:** ^1^Department of Pharmacology and Therapeutics, Faculty of Medicine, University of British Columbia, Vancouver, BC, Canada V6T 1Z4; ^2^Department of Physiology, Faculty of Medicine, Isfahan University of Medical Sciences, Isfahan, Iran

## Abstract

The aim of this study was to evaluate the effects of moderate-intensity exercise on plasma levels of C-reactive protein (CRP) and tumor necrosis factor-alpha (TNF-*α*) as markers of low-grade inflammation and endothelial function in diabetic (db/db) mice. Control and db/db mice were divided into sedentary and exercised groups. Aortic endothelial function was evaluated after two- and six-week exercises using a wire myograph. Plasma CRP levels were measured at baseline, and after two and six weeks of exercise. Baseline plasma CRP levels were significantly higher in db/db mice compared to control (*P* < .05). After two weeks of exercise, aortic endothelial function was significantly improved without affecting body weight or plasma CRP levels. Six weeks of exercise not only improved endothelial function, but also significantly reduced body weight and plasma CRP levels in db/db mice. Thus short-term exercise has beneficial effect on endothelial function without reducing low-grade inflammation while more prolonged exercise periods are required to reduce inflammatory markers.

## 1. Introduction

Cardiovascular diseases are the leading cause of morbidity and mortality in diabetic patients [1], and it is likely that vascular abnormalities may be responsible for the higher incidence of cardiovascular diseases in diabetes. Although it is suggested that endothelial dysfunction is an important contributor to the vascular complications of diabetes [2, 3], the exact mechanisms of impaired endothelial function are unclear. 

Lifestyle modification, especially exercise, is routinely recommended for the management of human type 2 diabetes [4, 5]. Exercise is thought to improve vascular function by reducing plasma lipids and blood glucose level [6], oxidative stress [7], and increasing insulin sensitivity [8]. Endothelial dysfunction is one of the earliest events in the progression of cardiovascular diseases.

Chronic low-grade inflammation, as reflected by elevated plasma levels of CRP, is an independent predictor of cardiovascular disease [9, 10] and diabetes [11]. CRP has a number of roles in several cardiovascular diseases [12], and levels of CRP are positively correlated with obesity and insulin resistance [13]. Many studies suggest that a chronic inflammatory process promotes the progression of endothelial dysfunction [14]. In this study, we hypothesized that moderate-intensity exercise improves endothelial function by decreasing low-grade inflammation in db/db mice, a frequently used animal model of type 2 diabetes. 

## 2. Materials and Methods

### 2.1. Animal Groups

Six-week-old control wild type and diabetic db/db mice (BKS.cg-m +/+ Lepr db/J) were purchased from Jackson Laboratory (Bar Harbor, ME, USA). All experiments were performed according to the guidelines of the University of British Columbia Animal Care Committee. After one week of acclimatization, animals were randomly divided into four groups (*n* = 10 each): two groups each of control (control sedentary and control exercised) and diabetic mice (diabetic sedentary, diabetic exercised). The animals were housed ten per cage under conditions of a 12-hour light/dark cycle, 22°C temperature, and with free access to food and water. Body weights were recorded weekly.

### 2.2. Exercise Program

Mice were exercised using a running wheel (Lafayette Instruments, Lafayette, IN, USA) as previously described in [2, 15]. Mice assigned to the exercise groups were placed in individual running wheels for one hour of daily exercise at a speed of 5.2 m/min (which represents a daily forced exercise of 312 m) for 6 weeks. During the training period (two weeks), mice were exercised daily at a set time each day for 5 days a week. The sedentary db/db or control groups were placed in nonrotating wheels for one hour per day. 

### 2.3. Plasma Variables

Animals were anaesthetized with pentobarbital (50 mg/kg, *i.p*.) combined with heparin (50 U/kg). Blood samples were taken at baseline (6 weeks old), after two weeks of exercise following a two-week training period (the 10th week) and at the end of study (the 14th week). Fasting blood glucose was measured using commercially available kits. Plasma CRP and TNF-*α* levels were measured using ELISA kits (Alpco Diagnostic, USA).

### 2.4. Evaluation of Endothelial Function

Thoracic aortas were removed and placed in ice-cold physiological salt solution (PSS) and cleaned of connective tissue. Segments of aorta were threaded with stainless steel wire (0.04 mm diameter) and attached to the tissue holders of a four-channel wire myograph (JP Trading, Aarhus, Denmark). Tissues were allowed to equilibrate for 60 minutes at 37°C during which time the PSS was replaced at 20-minute intervals. During the equilibration period, the resting tension was gradually increased to 5.5 mN and kept at this level for 20 to 30 minutes. Each tissue was maximally activated with a solution of KCl (80 mmol/L) that was prepared by equimolar substitution of NaCl. Following washout with fresh PSS and return of tension to basal preload, phenylephrine (1 *μ*mol/L) was added to establish a stable contraction. Thereafter, cumulative additions of acetylcholine (ACh) (1 nmol/L to 10 *μ*mol/L) were made. Vasodilatory responses were recorded on a computer using MyoDaq Acquisition software (version 2.01; Danish MyoTechnology, Aarhus, Denmark) and expressed as percent dilation of phenylephrine-induced constriction.

### 2.5. Citrate Synthase Assay

To document the efficacy of an endurance-trained state, citrate synthase activity levels were measured in skeletal muscle. Thigh adductor muscles were gently removed after sacrificing the animal, and citrate synthase activity was measured as previously described in [16]. 

### 2.6. Drugs and Chemicals

Acetylcholine, and phenylephrine were purchased from Sigma Chemical Co (St. Louis, MO). The composition of the PSS (mM) was NaCl (119), KCl (4.7), KH_2_PO_4_ (1.18), MgSO_4_ (1.17), NaHCO_3_ (24.9), EDTA (0.023), CaCl_2_ (1.6), and dextrose (11.1). Isotonic substitutions (replacement of Na^+^ with equimolar concentrations of K^+^) were used when using PSS solutions with increased K^+^ concentrations. 

### 2.7. Statistical Analysis

Results are expressed as mean ± SEM. Data analysis was done using NCSS-2000 software and GraphPad Prism (version 3.02-2000). ANOVA with multiple comparisons was performed using the Bonferroni's test. Correlation analysis using Spearman coefficient tests were performed where appropriate. *P* < .05 was considered as being statistically significant. 

## 3. Results

### 3.1. Body Weight, Blood Parameters, and Effect of Exercise

Six-week old diabetic mice had higher body weights than control mice. After six weeks of exercise, db/db exercised had lower body weights compared to the sedentary group (Table 1). Analysis of baseline blood parameters (6 weeks old), after two weeks (10 weeks old) and six weeks (14 weeks old) of exercise are shown in Table 1. Diabetic mice had higher fasting blood glucose levels at all time points, and while two weeks exercise did not alter blood glucose levels in db/db mice, six weeks of exercise reduced blood glucose levels in diabetic mice (*P* < .05). 

Baseline plasma CRP levels were higher in db/db mice compared to control (3.81 ± 0.23 versus 1.83 ± 0.30) (*P* < .05). Plasma CRP levels in db/db mice were not affected by two weeks of exercise but were significantly reduced after 6 weeks exercise (at the 14th week) (3.59 ± 0.41 versus 5.12 ± 0.25) (*P* < .05). Plasma levels of CRP were significantly correlated with body weight (*r* = 0.5855, *P* < .0001) and blood glucose (*r* = 0.4821, *P* = 0.0003) when analyzed by the Pearson test.

The level of plasma TNF-*α* in sedentary db/db mice at 14 weeks old (18.62 ± 2.11 pg/mL) tends to be higher than in control mice (14.88 ± 0.35 pg/mL); however, it does not reach statistical significance (*P* > .05).

### 3.2. Endothelial Function

**Figure 1:**
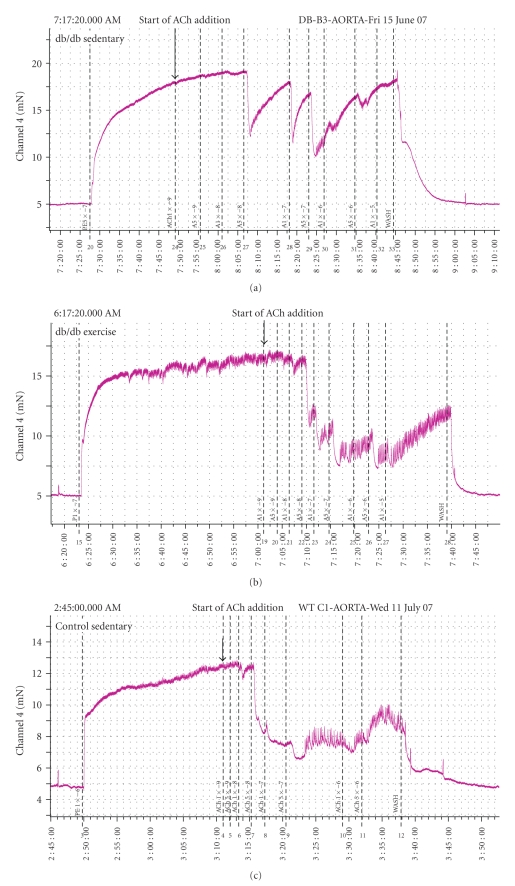

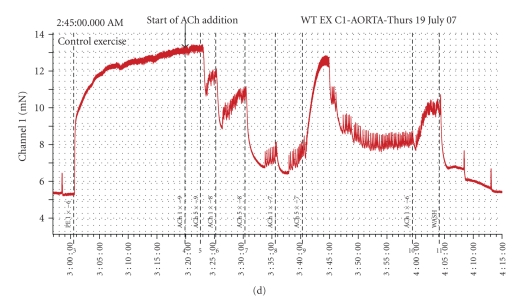
Representative traces showing ACh-induced vasodilation in aortae from diabetic (db/db) and control mice that were either sedentary or exercised.

**Figure 2:**
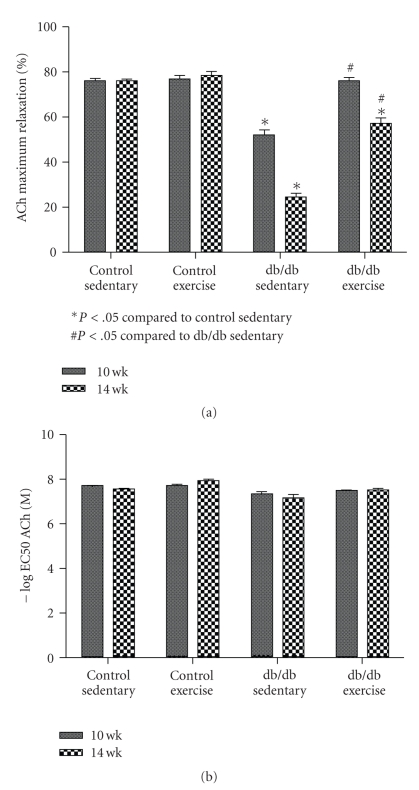
Emax (a) and EC50 (b) of ACh vasodilation after two weeks (10-week old mice) and six weeks (14-week old mice) of moderate-intensity exercise.

Acetylcholine (ACh) was used to evaluate endothelial-dependent vasodilatation. Responses to ACh vasodilation were impaired in aortic rings from db/db mice compared with control counterparts (Figure 1). Moderate-intensity exercise in db/db mice for either two or six weeks restored endothelium-dependent vasodilation (Figure 1). The maximal vasodilatation (% loss of induced tone) and sensitivity (EC_50_) is shown in Figure 2. 

### 3.3. Citrate Synthase Activity

As shown in Table 2, tissue levels of citrate synthase activity were significantly increased in the thigh adductor muscles of db/db and control exercised mice compared to the sedentary groups at both time points (after two and six weeks of exercise) (*P* < .01, *n* = 10). 

## 4. Discussion

This study examined the effects of moderate levels of exercise on vascular endothelial function and plasma CRP levels in control and type 2 diabetic (db/db) mice. We report that endothelial function (endothelium-dependent relaxation) was significantly impaired in db/db mice, as also reported in other studies [17–19]. There is much evidence to support the notion that endothelial dysfunction precedes the development of type 2 diabetes [20, 21]. Two-week and six-week of moderate-intensity exercise both significantly improved endothelium-dependent relaxation in db/db mice.

There is a strong association between endothelial dysfunction and inflammation. Endothelial dysfunction and plasma markers of inflammation are consistently increased in type 2 diabetes [22]. Our results show that diabetic mice initially have higher CRP levels compared to control animals. An association between CRP levels and diabetes has been reported in other studies. For example, plasma levels of plasma CRP and ICAM levels are higher in diabetic subjects [23–25], and it is likely that increases in CRP levels also occur in patients with impaired glucose tolerance [26]. Thus, hyperglycemia may be one reason for endothelial dysfunction and low-grade inflammation in db/db mice [27]. Hyperglycemia is thought to activate the immune and macrophage-monocyte systems and so stimulate the production of cytokines and acute phase proteins, which are also proposed to reduce endothelial dependent vasodilation [22, 28]. Moreover, highly-glycated haemoglobin impairs NO-mediated vascular responses by a mechanism involving superoxide anions but not cyclooxygenase derivatives [7, 29]. In addition, db/db mice are obese, and there is also a close association between adiposity and CRP [13, 30]. Adipose tissue secretes inflammatory mediators (especially IL-6) which stimulates CRP synthesis in the liver [31]. CRP is related to insulin resistance and is a marker of endothelial dysfunction [32]. 

In our experiments, exercise improved endothelium-dependent relaxation in db/db mice after two-week exercise independently of reductions in weight, blood glucose, or plasma CRP levels; our data shows a lack of a correlation between improved vasodilatation to ACh and decreased plasma CRP levels after two weeks of exercise as shown by the nonsignificant (*P* = .1941) Pearson correlation coefficient for the relationship between maximal ACh dilation and plasma CRP levels. However, six weeks of exercise improved ACh-mediated vasodilatation while also reducing plasma CRP levels in db/db mice; this was associated with a significant correlation between plasma CRP levels and body weight, a finding that is consistent with other reports in experimental [33] and human diabetes [34]. 

Our results indicate that CRP levels are increased in control mice that underwent a period of forced-exercise. This finding is in keeping with recent studies in healthy humans indicating that there were significant increases in plasma CRP and TNF alpha following a two-week bout of exercise [35]. In addition, exercise has also been shown to stimulate a marked but transient increases in inflammatory markers such as IL-6 and cortisol (which subsequently stimulate hepatocytes to generate the synthesis of acute phase proteins such as CRP), responses that may reflect muscle injury [36, 37]. 

Since CRP can a cause dose-dependent decrease in NO production in endothelial cells [38], it is possible that this effect is time-dependent and occurs independently of inflammation as reported by CRP levels. Other studies have reported that that CRP directly inhibits the endothelium-dependent NO mediated dilation of porcine retinal arterioles [39], and down-regulates eNOS protein to decrease NO release [40].

The plasma levels of TNF-*α* in sedentary db/db mice tends to be higher than in control mice; however, it does not reach statistical significance. Previous reports have failed to demonstrate a parallelism between changes in plasma levels of CRP, IL-6, and TNF-*α* under pathological conditions [41–43]. Overweight adolescent boys had higher TNF-*α*, but not CRP or IL-6 levels compared to normal weight controls [42]. A systematic review demonstrated that exercise decreases CRP with no apparent effects on TNF-*α* [41]. However, CRP is the marker of chronic inflammation most frequently studied [44] and has been shown to predict cardiovascular diseases more than other cytokines [45].

In conclusion, we report a reciprocal association between endothelial dysfunction and CRP levels in diabetic db/db mice. Short-term exercise improves endothelial function without changing plasma CRP levels (two weeks of exercise). Longer periods of exercise (six weeks) reduce plasma CRP levels and maintain improved endothelial function in diabetic mice. 

## Figures and Tables

**Table 1:** Body weights, plasma glucose CRP, and TNF-*α* levels in control or diabetic (db/db) mice that were either sedentary or exercised.

	Control sedentary			Control exercise			db/db Sedentary		db/db exercise	
	6 wk	10 wk	14 wk	10 wk	14 wk	6 wk	10 wk	14 wk	10 wk	14 wk
Body weight (gm)	20.7 ± 0.3	28.0 ± 0.3	31.9 ± 0.4	25.8 ± 0.4	28.3 ± 0.6*	30.7 ± 0.4^#^	45.9 ± 0.7^#^	48.9 ± 1.2^#^	43.6 ± 1.0^#^	44.6 ± 1.3^∗#^

Fasting blood glucose (mg/dL)	2.3 ± 0.1	5.0 ± 0.2	5.9 ± 0.5	5.3 ± 0.2	5.8 ± 0.2	5.7 ± 0.3^#^	31.5 ± 1.3^#^	54.7 ± 1.5^#^	30.3 ± 1.9^#^	44.6 ± 1.6^∗#^

Plasma CRP (ng/mL)	1.8 ± 0.3	2.6 ± 0.3	2.5 ± 0.4	3.8 ± 0.2*	4.5 ± 0.3*	3.3 ± 0.3^#^	4.1 ± 0.2^#^	5.1 ± 0.3^#^	3.8 ± 0.4^#^	3.6 ± 0.4*

PlasmaTNF-*α* (pg/mL)	N/A	N/A	14.88 ± 0.35	N/A	14.30 ± 0.74	N/A	N/A	18.62 ± 2.11	N/A	20.53 ± 1.85

*P < .05 compared to sedentary group at the same age.

^#^ *P* < .05 compared to control groups.

N/A: variable not measured

**Table 2:** Citrate synthase activity (umole/mL/min) in thigh adductor muscle of all experimental groups.

	Control sedentary	Control exercised	db/db sedentary	db/db exercised
(10 Week old)	4.7 ± 0.053	5.0 ± 0.064*	3.6 ± 0.058	3.9 ± 0.042*
(14 Week old)	4.21 ± 0.32	6.61 ± 0.54*	1.67 ± 0.18	2.16 ± 0.12*

*P < .05 compared to sedentary group.

## References

[1] Garcia MJ, McNamara PM, Gordon T, Kannell WB (1974). Morbidity and mortality in diabetics in the Framingham population. Sixteen year follow up study. *Diabetes*.

[2] Khazaei M, Moien-Afshari F, Kieffer TJ, Laher I (2008). Effect of exercise on augmented aortic vasoconstriction in the db/db mouse model of type-II diabetes. *Physiological Research*.

[3] Moien-Afshari F, Ghosh S, Elmi S (2008). Exercise restores coronary vascular function independent of myogenic tone or hyperglycemic status in *db/db* mice. *American Journal of Physiology*.

[4] Myers J, Prakash M, Froelicher V, Do D, Partington S, Edwin Atwood J (2002). Exercise capacity and mortality among men referred for exercise testing. *The New England Journal of Medicine*.

[5] Paffenbarger RS, Hyde RT, Wing AL, Lee I-M, Jung DL, Kampert JB (1993). The association of changes in physical-activity level and other lifestyle characteristics with mortality among men. *The New England Journal of Medicine*.

[6] Knowler WC, Barrett-Connor E, Fowler SE (2002). Reduction in the incidence of type 2 diabetes with lifestyle intervention or metformin. *The New England Journal of Medicine*.

[7] Ghosh S, Khazaei M, Moien-Afshari F (2009). Moderate exercise attenuates caspase-3 activity, oxidative stress, and inhibits progression of diabetic renal disease in *db/db* mice. *American Journal of Physiology*.

[8] Boulé NG, Haddad E, Kenny GP, Wells GA, Sigal RJ (2001). Effects of exercise on glycemic control and body mass in type 2 diabetes mellitus: a meta-analysis of controlled clinical trials. *Journal of the American Medical Association*.

[9] Cesari M, Penninx BWJH, Newman AB (2003). Inflammatory markers and onset of cardiovascular events: results from the health ABC study. *Circulation*.

[10] Cesari M, Penninx BWJH, Newman AB (2003). Inflammatory markers and cardiovascular disease (The Health, Aging and Body Composition [Health ABC] Study). *American Journal of Cardiology*.

[11] Schmidt MI, Duncan BB, Sharrett AR (1999). Markers of inflammation and prediction of diabetes mellitus in adults (Atherosclerosis Risk in Communities study): a cohort study. *The Lancet*.

[12] Verma S, Szmitko PE, Ridker PM (2005). C-reactive protein comes of age. *Nature Clinical Practice Cardiovascular Medicine*.

[13] Visser M, Bouter LM, McQuillan GM, Wener MH, Harris TB (1999). Elevated C-reactive protein levels in overweight and obese adults. *Journal of the American Medical Association*.

[14] Ross R (1999). Atherosclerosis—an inflammatory disease. *The New England Journal of Medicine*.

[15] Moien-Afshari F, Ghosh S, Khazaei M, Kieffer TJ, Brownsey RW, Laher I (2008). Exercise restores endothelial function independently of weight loss or hyperglycaemic status in *db/db* mice. *Diabetologia*.

[16] Korzick DH, Laughlin MH, Bowles DK (2004). Alterations in PKC signaling underlie enhanced myogenic tone in exercise-trained porcine coronary resistance arteries. *Journal of Applied Physiology*.

[17] Enderle M-D, Benda N, Schmuelling R-M, Haering HU, Pfohl M (1998). Preserved endothelial function in IDDM patients, but not in NIDDM patients, compared with healthy subjects. *Diabetes Care*.

[18] Goodfellow J, Ramsey MW, Luddington LA (1996). Endothelium and inelastic arteries: an early marker of vascular dysfunction in non-insulin dependent diabetes. *British Medical Journal*.

[19] Makimattila S, Liu M-L, Vakkilainen J (1999). Impaired endothelium-dependent vasodilation in type 2 diabetes: relation to LDL size, oxidized LDL, and antioxidants. *Diabetes Care*.

[20] Caballero AE, Arora S, Saouaf R (1999). Microvascular and macrovascular reactivity is reduced in subjects at risk for type 2 diabetes. *Diabetes*.

[21] Balletshofer BM, Rittig K, Enderle MD (2000). Endothelial dysfunction is detectable in young normotensive first-degree relatives of subjects with type 2 diabetes in association with insulin resistance. *Circulation*.

[22] Calles-Escandon J, Cipolla M (2001). Diabetes and endothelial dysfunction: a clinical perspective. *Endocrine Reviews*.

[23] Nyström T, Nygren A, Sjöholm Å (2005). Persistent endothelial dysfunction is related to elevated C-reactive protein (CRP) levels in Type II diabetic patients after acute myocardial infarction. *Clinical Science*.

[24] Schalkwijk CG, Poland DCW, van Dijk W (1999). Plasma concentration of C-reactive protein is increased in Type I diabetic patients without clinical macroangiopathy and correlates with markers of endothelial dysfunction: evidence for chronic inflammation. *Diabetologia*.

[25] Targher G, Bertolini L, Zoppini G, Zenari L, Falezza G (2005). Increased plasma markers of inflammation and endothelial dysfunction and their association with microvascular complications in Type 1 diabetic patients without clinically manifest macroangiopathy. *Diabetic Medicine*.

[26] Henareh L, Jogestrand T, Agewall S (2005). Glucose intolerance is associated with C-reactive protein and intima-media anatomy of the common carotid artery in patients with coronary heart disease. *Diabetic Medicine*.

[27] Gómez JM, Vila R, Catalina P, Soler J, Badimón L, Sahún M (2008). The markers of inflammation and endothelial dysfunction in correlation with glycated haemoglobin are present in type 2 diabetes mellitus patients but not in their relatives. *Glycoconjugate Journal*.

[28] Pankewycz OG, Guan J-X, Benedict JF (1995). Cytokines as mediators of autoimmune diabetes and diabetic complications. *Endocrine Reviews*.

[29] Vallejo S, Angulo J, Peiró C (2000). Highly glycated oxyhaemoglobin impairs nitric oxide relaxations in human mesenteric microvessels. *Diabetologia*.

[30] Kopp HP, Kopp CW, Festa A (2003). Impact of weight loss on inflammatory proteins and their association with the insulin resistance syndrome in morbidly obese patients. *Arteriosclerosis, Thrombosis, and Vascular Biology*.

[31] Fain JN, Madan AK, Hiler ML, Cheema P, Bahouth SW (2004). Comparison of the release of adipokines by adipose tissue, adipose tissue matrix, and adipocytes from visceral and subcutaneous abdominal adipose tissues of obese humans. *Endocrinology*.

[32] Savoia C, Schiffrin EL (2007). Vascular inflammation in hypertension and diabetes: molecular mechanisms and therapeutic interventions. *Clinical Science*.

[33] de Lemos ET, Reis F, Baptista S (2007). Exercise training is associated with improved levels of C-reactive protein and adiponectin in ZDF (type 2) diabetic rats. *Medical Science Monitor*.

[34] Wong PCH, Chia MYH, Tsou IYY (2008). Effects of a 12-week exercise training programme on aerobic fitness, body composition, blood lipids and C-reactive protein in adolescents with obesity. *Annals of the Academy of Medicine Singapore*.

[35] Andersson J, Jansson J-H, Hellsten G, Nilsson TK, Hallmans G, Boman K (2010). Effects of heavy endurance physical exercise on inflammatory markers in non-athletes. *Atherosclerosis*.

[36] Chatzinikolaou A, Fatouros IG, Gourgoulis V (2010). Time course of changes in performance and inflammatory responses after acute plyometric exercise. *Journal of Strength and Conditioning Research*.

[37] Ispirlidis I, Fatouros IG, Jamurtas AZ (2008). Time-course of changes in inflammatory and performance responses following a soccer game. *Clinical Journal of Sport Medicine*.

[38] Verma S, Wang C-H, Li S-H (2002). A self-fulfilling prophecy: C-reactive protein attenuates nitric oxide production and inhibits angiogenesis. *Circulation*.

[39] Nagaoka T, Kuo L, Ren Y, Yoshida A, Hein TW (2008). C-reactive protein inhibits endothelium-dependent nitric oxide-mediated dilation of retinal arterioles via enhanced superoxide production. *Investigative Ophthalmology and Visual Science*.

[40] Steffens S, Mach F (2004). Inflammation and atherosclerosis. *Herz*.

[41] de Salles BF, Simao R, Fleck SJ (2010). Effects of resistance training on cytokines. *International Journal of Sports Medicine*.

[42] MacEneaney OJ, Harrison M, O’Gorman DJ, Pankratieva EV, O’Connor PL, Moyna NM (2009). Effect of prior exercise on postprandial lipemia and markers of inflammation and endothelial activation in normal weight and overweight adolescent boys. *European Journal of Applied Physiology*.

[43] Palmer-Kazen U, Religa P, Wahlberg E (2009). Exercise in patients with intermittent claudication elicits signs of inflammation and angiogenesis. *European Journal of Vascular and Endovascular Surgery*.

[44] Nicklas BJ, You T, Pahor M (2005). Behavioural treatments for chronic systemic inflammation: effects of dietary weight loss and exercise training. *Canadian Medical Association Journal*.

[45] de Ferranti S, Rifai N (2002). C-reactive protein and cardiovascular disease: a review of risk prediction and interventions. *Clinica Chimica Acta*.

